# The Evansville Dental Society

**Published:** 1897-12

**Authors:** 


### The Evansville Dental Society

HAS A MEETING FOR SOCIAL AND PROFESSIONAL BENEFIT—ADOPTS RESOLUTIONS IN HONOR OF A DEPARTING MEMBER —A NUMBER OF INTERESTING AND INSTRUCTIVE PAPERS READ —EVENING CONCLUDES WITH A BANQUET.

A very interesting meeting of the Evansville Dental Society, was held July 20th, in the evening, at the office of Dr. C. E. Pittman, all the members being present. Dr. Gilmore, of Princeton; Dr. Hale of Mt. Vernon and Dr. Meade of Carmi, Ills., were guests of the society. Interesting papers were read by the following gentlemen: Dr. S. F. Gilmore, subject, "Cataphoresis;" Dr. S. B. Lewis, subject, "Plastics;" Dr. C. Chandler George, subject, " A Plea for the Retention of the First Permanent Molars."

The following resolutions were adopted by the society in honor of Dr. S. F. Jacobi, who is soon to remove to Selma, Ala.:

" Be it resolved by all the members present, that insomuch as our friend and co-laborer, Dr. S. F. Jacobi, has tendered his resignation as a member of this society, he being soon to depart to a new field of lab r, that in accepting his resignation we do so with the fullest sense of regret at being obliged to lose from our circle one who has aided us by his counsel and in every way sought by his professional conduct to upbuild and sustain the profession of dentistry in our midst, and that now upon severing his connection we manifest to him our hearty approval of his friendship, and thus assure him of the fact that we as his professional brothers and friends wish him God's choicest blessings and a full measure of the world's best honors, and that there may be meted out to him in his new home an abundance of deserved success, is the wish of all the members of this society."

The committee appointed to draft the resolutions presented the doctor with an embossed copy, the pen work of Prof. Ellis, which was highly ornamental. The doctor being taken completely by surprise, endeavored to reply amid feelings of emotion.

At the conclusion of the discussion of the papers, the members present repaired to the Stag Hotel, where a banquet was served in honor of the departing member and the society's guests.

This meeting marks an epoch in the history of the society which will long be remembered as a social event, and tends to more securely cement the bonds of professional friendship. The society is in a prosperous condition and numbers among its members the following dentists of the city: F. Hutchinson, F. J. Raymond, C. Chandler George, C. E. Pittman, W. G. Downs, M. M. Haas, S. Clyde Smith, Emil Knapp, M. H. Martin, S. B. Lewis, W. E. Barter.

